# Design, Synthesis and Properties of Semi-Alicyclic Colorless and Transparent Polyimide Films with High Glass Transition Temperatures and Low Retardation for Potential Applications in Flexible Electronics

**DOI:** 10.3390/polym15163408

**Published:** 2023-08-14

**Authors:** Xi Ren, Zhibin He, Zhenzhong Wang, Zhen Pan, Yuexin Qi, Shujun Han, Haifeng Yu, Jingang Liu

**Affiliations:** 1 Engineering Research Center of Ministry of Education for Geological Carbon Storage and Low Carbon Utilization of Resources, School of Materials Science and Technology, China University of Geosciences, Beijing 100083, China; renxi@email.cugb.edu.cn (X.R.); wzz0808@163.com (Z.W.); 2103210036@email.cugb.edu.cn (Z.P.); qiyuexin1004@cugb.edu.cn (Y.Q.); 15966200097@163.com (S.H.); 2 School of Material Science and Engineering, Key Laboratory of Polymer Chemistry and Physics of Ministry of Education, Peking University, Beijing 100871, China; zb.he@stu.pku.edu.cn (Z.H.); yuhaifeng@pku.edu.cn (H.Y.)

**Keywords:** alicyclic polyimide, low retardation, fluorene, fluorine, optical properties, thermal properties

## Abstract

Polyimide (PI) optical films with high glass transition temperatures (high-T_g_), high optical transparency, and low optical retardations (low-R_th_) are highly desired in advanced optoelectronic applications. However, the standard PI films usually suffer from deep colors, high optical anisotropies and limited T_g_ values. In the current work, a series of semi-alicyclic colorless and transparent PI (CPI) films were developed from hydrogenated pyromellitic dianhydride stereoisomers, 1S,2R,4S,5R-hydrogenated pyromellitic dianhydride and 1R,2S,4S,5R-hydrogenated pyromellitic dianhydride, and fluorene-containing diamines, including 9,9-bis(4-aminophenyl)fluorene and 9,9-bis(3-fluoro-4-aminophenyl)fluorene, respectively. The derived CPI films showed T_g_ values higher than 420 °C according to differential scanning calorimetry measurements. In addition, the fluorene-based CPI film showed optical transmittances higher than 80% at the wavelength of 400 nm, with yellow indices in the range of 0.60~1.01 and haze values below 3.0%. The CPI films showed average refractive indices from 1.5407 to 1.6309, extremely low birefringence at the level of minus fourth power of ten, and further exhibited quite low optical retardations below 10 nm.

## 1. Introduction

Applications as optical components in various high-tech devices have been becoming one of the most important hot topics in relation to the research and development of high-performance polyimide (PI) films [1,2,3,4,5]. In practical optical applications of the PI films, such as optical fiber communications, optical waveguides, liquid crystal display and rigid or flexible organic light-emitting diode displays, adjustments of the optical parameters of PI films, including optical transparency, color parameters, refractive indices (n), birefringence (Δn), optical retardations and so on, play important roles for achieving the desirable performance of the optical devices [6]. In particular, in flexible electronics areas, including flexible display, flexible solar cells, flexible electronic skins, and flexible and wearable devices, the optical properties of PI films have to be elaborately tailored so as to meet the different property requirements [7,8,9]. Among the various optical properties of the PI films, the adjustments of the optical retardations (R_th_) of the films have been paid increasing attention in recent years. For example, in a flexible active matrix organic light-emitting diode (AMOLED) display device, the optical film used as a cover window needs to have the lowest optical anisotropy possible in order to reduce the display defects [10]. However, in thin film transistor-driven active matrix liquid crystal display (TFT-LCD) devices, some optical films should possess high optical anisotropies [11]. In view of the potential ability to eliminate display nonuniformities in the cover window films for flexible AMOLED, polymeric optical films with low R_th_ (low-R_th_) and high thermal stability features are highly desired [12].

It has been well established that the R_th_ values of polymer films are mostly related to the chemical structures and fabrication processes. The R_th_ parameter is usually expressed via the equation R_th_ = Δn × d, where Δn stands for the birefringence of the polymer film and d represents the thickness of the film. Some polymers with rigid structures show high birefringence after casting the film on substrates. Standard aromatic PI films, such as the unstretched species derived from pyromellitic dianhydride (PMDA) and 4,4′-oxydianiline (ODA) (PI_PMDA-ODA_), and the ones from rigid-rod 3,3′,4,4′-biphenyl- enetetracarboxylic acid dianhydride (BPDA) and para-phenylenediamine (PDA) (PI_BPDA-PDA_), showed high Δn values of 0.077 and 0.22 at a thickness around 20 μm, respectively [13], indicating R_th_ values over thousands of nanometers. This is usually unacceptable for specific optical applications. In addition, the fabricating conditions, including the uniaxial stretching [14,15,16], biaxial stretching [17], drying or imidization procedures [18], and other factors, might severely affect the optical anisotropies of the derived PI films. Excluding the influence of processing conditions on the optical anisotropies of the PI films, the molecular structures of PI films are also important factors affecting the optical anisotropies. It was reported that PI films with rigid molecular skeletons, such as PI_BPDA-ODA,_ usually show relatively high R_th_ values, while PI films with less rigid or flexible units, such as the ones containing flexible ether linkages, isopropyl or hexafluoroisopropyl groups, and so on, might possess lower R_th_ values. What is more, the PI films derived from preimidized and organo-soluble resins, such as the ones based on 3,3′,4,4′-benzophenonetetracarboxylic acid dianhydride (BTDA) and 5(6)-amino-1-(4-aminophenyl)-1,3,3-trimethylindane (PIDA) (PI_BTDA-PIDA_), usually showed much lower optical anisotropies compared with those derived from the poly(amic acid) (PAA) precursors due to the potentially increasing optical anisotropies in the films during the high-temperature imidization process [19]. Generally speaking, in order to develop PI films with low optical anisotropies or low retardation features, resins with preimidized structural characteristics together with flexible molecular skeletons are highly preferred [20]. Recently, Li and coworkers reported colorless PI films with optical retardation as low as 42 nm at the thickness of 21 μm achieved by the polymerization of flexible 4,4′-(4,4′-isopropylidenediphenoxy)bis(phthalic anhydride) (BPADA) and bis[4-(3-aminophenoxy)phenyl]sulfone (mBAPS) [21]. However, the derived PI_BPADA-mBAPS_ film showed low glass transition temperatures (T_g_) of 200 °C and 224 °C measured by differential scanning calorimetry (DSC) and dynamic mechanical analysis (DMA) methods, respectively.

It is well known that fluorene-based polymers usually have a high refractive index (n) and low birefringence (Δn); thus they have been widely used to develop optical components with extremely low optical retardation, such as optical microlenses for digital cameras and so on [22,23,24,25]. Meanwhile, bulky fluorene groups could usually endow the polymers with high thermal stability due to the restricted internal rotations around the fluorene units, for which a large sweep volume is usually required for the free rotational motion [26]. On the other hand, alicyclic units have also been widely used to develop polymer optical films with low R_th_ characteristics. Polymer films containing alicyclic units, such as the cyclo-olefin polymers (COPs), could achieve almost zero retardation and an optical transmittance over 90% in the visible light region (400~700 nm) [27]. The semi-alicyclic PI films derived from the alicyclic dianhydride of CpODA and the aromatic diamine of 3,4′-oxydianiline (3,4′-ODA) exhibited low R_th_ values of 1.7 nm [28]. However, PI_CpODA-3,4′-ODA_ could only be fabricated by the two-step high-temperature imidization procedure via the PAA precursor.

Based on the above-mentioned analyses about the effects of structure on the R_th_ features of the optical polymer films, one can deduce that high-performance colorless and transparent PI (CPI) films might be developed via simultaneously incorporating alicyclic and fluorene moieties into the PI structures. Thus, in the current work, a series of solution-processable semi-alicyclic CPI films were developed from the preimidized resins based on the alicyclic dianhydride of hydrogenated PMDA (HPMDA) and the fluorene-containing aromatic diamines. The effects of the alicyclic and fluorene units on the thermal, optical, and especially retardation properties of the derived CPI films were studied in detail.

## 2. Materials and Methods

### 2.1. Materials

1S,2R,4S,5R-hydrogenated pyromellitic dianhydride (ccHPMDA, I, melting point: 303.3 °C, DSC peak temperature) and 1R,2S,4S,5R-hydrogenated pyromellitic dianhydride (ctHPMDA, II, melting point: 273.1 °C, DSC peak temperature) were purchased from Newera Kesense New Materials Co., Ltd. (Weihai, China) and further purified in our laboratory, and then dried in vacuo overnight at 180 °C before use. 9,9-Bis(4-aminophenyl)fluorene (FDA) and 9,9-bis(3-fluoro-4-aminophenyl)fluorene (FFDA) were purchased from Tokyo Chemical Industry Co., Ltd. (Tokyo, Japan) and used as received. 2,2′-Bis(trifluoromethyl)-4,4′-diamino diphenylether (6FODA) was purchased from Chinatech Chem. Co., Ltd. (Tianjin, China) and dried at 80 °C in vacuo for 24 h prior to use. The anhydrous γ-butyrolactone (GBL) and N,N-dimethylacetamide (DMAc) were purchased from Innochem Science & Technology Co., Ltd. (Beijing, China) and used directly. The other chemicals were obtained from Sinopharm Chemical Reagent Co., Ltd. (Shanghai, China) and were used as received.

### 2.2. Characterization Methods

An Ubbelohde viscometer (Sogo Laboratory Glass Works Co., Ltd., Kyoto, Japan) was used to measure the inherent viscosities of the PI resins using a 0.5 g/dL NMP solution at 25 °C. The number average molar mass (M_n_) and weight average molar mass (M_w_) of the PI resins were tested with a gel permeation chromatography (GPC) system (Shimadzu, Kyoto, Japan) with the HPLC grade of N-methyl-2-pyrrolidone (NMP) as the mobile phase. Hydrogen nuclear magnetic resonance (^1^H-NMR) spectra of the PI resins were recorded on a picoSpin 45 spectrometer (Thermo Fisher Scientific, San Jose, CA, USA) in DMSO-d_6_. The Fourier transform infrared (FTIR) spectra of the CPI films were detected with a Perkin-Elmer 1600 Series FTIR spectrometer (Perkin-Elmer Inc., Norwalk, CT, USA). Wide-angle X-ray diffraction (XRD) of the CPI films was conducted on a Rigaku D/max-2500 X-ray diffractometer (Tokyo, Japan) operated at 40 kV and 200 mA. Ultraviolet–visible (UV-Vis) spectra of the CPI films were measured on a Shimadzu UV-1800 spectrophotometer (Shimadzu Inc. Kyoto, Japan) at room temperature. The in-plane refractive indices (n_TE_), and out-of-plane refractive indices (n_TM_) of the CPI films were measured with a Metricon Model 2010/M prism coupler (Pennington, NJ, USA) at the wavelength of 632.8 nm. The average refractive indices (n_av_) were calculated as n_av_ = [(2n_TE_^2^ + n_TM_^2^)/3]^1/2^. The birefringence (Δn) values of the CPI films were calculated as Δn = n_TE_ − n_TM_ and the R_th_ values were calculated as R_th_ = Δn × d, where d stands for the thickness of the CPI films.

The CIE (International Commission on Illumination) color parameters of the CPI films, including the L*, a* and b*, were measured using an X-rite color i7 spectrophotometer (Grand Rapids, MI, USA) at a thickness of 50 μm. Thermogravimetric analysis (TGA) and the derivative TGA (DTG) of the CPI films were performed on a TG 209 F3 thermogravimetric analyzer (Netzsch, Selb, Germany) at a heating rate of 20 °C/min in nitrogen. Differential scanning calorimetry (DSC) was carried on a DSC 3500 thermal analysis system (Netzsch, Selb, Germany) at a heating rate of 10 °C/min in nitrogen. Dynamic mechanical analysis (DMA) was recorded on a TA-Q800 thermal analysis system (New Castle, DE, USA) at a heating rate of 5 °C/min and a frequency of 1 Hz in nitrogen. The thermo-mechanical analysis (TMA) was recorded on a TMA402F3 thermal analysis system (NETZSCH, Selb, Germany) with the temperatures ranging from 50 to about 450 °C at a heating rate of 5 °C/min in nitrogen atmosphere. The coefficients of the linear thermal expansion (CTE) values of composite films were recorded in the range of 50~250 °C.

The solubility values of the PI resins in the tested solvents were measured as follows. To a 100 mL flask we added 1.0 g of the PI resins and 9.0 g of the solvent tested to afford a mixture with the solid content of 10 wt. %. The mixture was magnetically stirred at room temperature for 24 h. Then, the solubility of the resin was determined with three grades: completely soluble (++), partially soluble (+), and insoluble (−).

### 2.3. CPI Resins Synthesis and Films Preparation

Four CPI resins, including the ones derived from ccHPMDA (I) and FDA (a) (CPI-I_a_), from ccHPMDA (I) and FFDA (b) (CPI-I_b_), from ctHPMDA (II) and FDA (a) (CPI-II_a_), and from ctHPMDA (I) and FFDA (b) (CPI-II_b_), were prepared according to the formula shown in Table 1. CPI-I_a_ (ccHPMDA-FDA) was used to show the detailed synthesis procedure. Into a 1000 mL three-necked flask settled in a thousand-class clean room we added anhydrous GBL (100 g) and FDA (34.844 g, 0.1 mol). Dry nitrogen was passed through the diamine solution. After stirring at room temperature for 10 min, the pale-brown FDA solution was obtained. Then, ccHPMDA (22.417 g, 0.1 mol) dianhydride was added into the reaction system with one batch via the adding funnel. Additional GBL (71.8 g) was added, and we washed all of the dianhydride into the reaction mixture. The stirring speed was then increased from 150 rpm to 300 rpm until a viscous solution was obtained. A moderate heat-releasing phenomenon was detected. Then the mixing speed was decreased to 200 rpm. Toluene (200 g) and isoquinoline (0.5 g) were added to the reaction system, after which the reaction mixture was heated under nitrogen protection. When the temperature of the reactant reached 130~140 °C, the toluene-water azeotrope was found to be trapped in the Dean–Stark trap. The water by-products were continuously distilled out of the reaction mixture to promote increases in the molecular weights of the resin. No water was observed after 6 h and the residual toluene was distilled out the system until the inner temperature of the reactant reached 180 °C. The reaction was maintained at this temperature for another 6 h and then cooled to room temperature. A viscous solution with a pale reddish-brown appearance was obtained. The solution was slowly added into the aqueous ethanol solution (70 vol%) to form threadlike precipitants. The resin was immersed into the ethanol solution for 24 h and collected. The resin was first dried in an air-circulating environment and then transferred into a vacuum-drying oven and dried in vacuo at 120 °C overnight. Lastly, the CPI-I_a_ resin was obtained in the form of flexible and tough silky solids. Yield: 51.9 g (96.7%). Numeric average molar mass (M_n_): 8.11 × 10^4^ g/mol. Weight average molar mass (M_w_): 1.29 × 10^5^ g/mol. Polydispersity index (PDI): 1.59. ^1^H-NMR (DMSO-d_6_, ppm): 7.99–7.96 (d, 2H), 7.52–7.17 (m, 10H), 3.19–3.12 (m, 4H), 2.28–2.11 (m, 2H), and 1.89–1.85 (m, 2H).

The dried CPI-I_a_ resin was mixed with anhydrous DMAc at a solid content of 25 wt. %. The mixture was stirred at room temperature for 10 h and the obtained homogeneous solution was filtered through a 1.0 μm Teflon syringe filter to remove any undissolved impurities. The purified CPI-I_a_ solution was then spin-coated on a 4-inch silicon wafer substrate. The thickness of the final films was controlled by adjusting the spinning speed of the apparatus. The substrate was then thermally baked on a heat plate with the procedure of 80 °C/2 h, 120 °C/1 h, 180 °C/1 h, and 250 °C/1 h nitrogen. After cooling to room temperature, the substrate was immersed into deionized water and the CPI-I_a_ film automatically peeled off from the substrate. The film was dried at 120 °C in vacuo before various property evaluations. FTIR (cm^−1^): 2935, 1780, 1705, 1508, 1373, 1180, and 748.

The other resins and films were prepared according to a similar procedure to that mentioned above with the formula shown in Table 1.

CPI-I_b_ (ccHPMDA-FFDA). FTIR (cm^−1^): 2935, 1788, 1713, 1508, 1385, 1178, and 742.

CPI-II_a_ (ctHPMDA-FDA). FTIR (cm^−1^): 2939, 1780, 1703, 1508, 1377, 1171, and 748.

CPI-II_b_ (ctHPMDA-FFDA). FTIR (cm^−1^): 2933, 1784, 1711, 1508, 1380, 1174, and 742.

Two CPI resins and films, including CPI-ref1 (ccHPMDA-6FODA) and CPI-ref2 (ctHPMDA-6FODA), were also prepared for reference according to a procedure similar to that mentioned above.

CPI-ref1 (ccHPMDA-6FODA). FTIR (cm^−1^): 2941, 1786, 1707, 1489, 1385, 1119, 1051, and 769.

CPI-ref2 (ctHPMDA-6FODA). FTIR (cm^−1^): 2937, 1786, 1709, 1489, 1385, 1119, 1051, and 769.

## 3. Results and Discussion

### 3.1. CPI Resins Synthesis and Films Preparation

**Figure 1:**
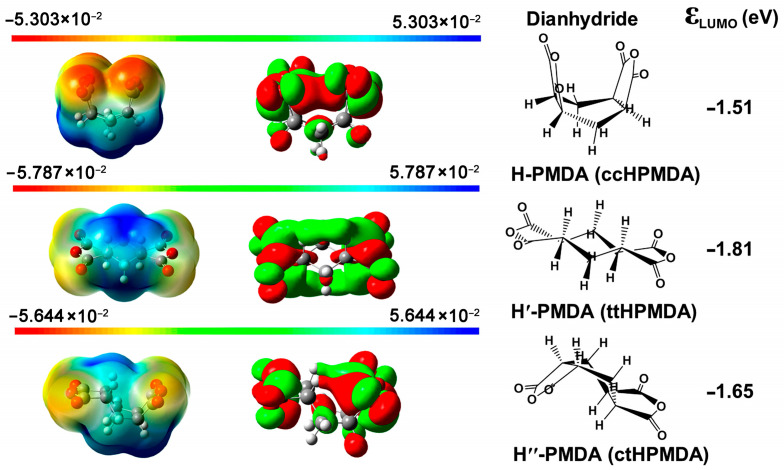
Molecular orbit energy levels (ε_LUMO_) of the HPMDA dianhydrides.

**Figure 2:**
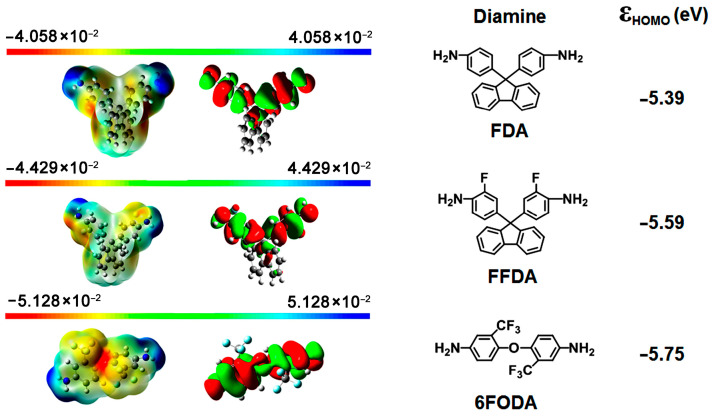
Molecular orbit energy levels (ε_HOMO_) of the aromatic diamines.

Two alicyclic dianhydrides with different steric structures, ccHPMDA and ctHPMDA, were used for the development of low-R_th_ and high-T_g_ CPI films in the current work. These series of hydrogenated pyromellitic dianhydrides (HPMDA) or cyclohexane dianhydrides (CHDA) have been thoroughly studied in Hasegawa’s pioneering works [29,30,31], and the effects of stereoisomerism in the dianhydride on the polymerization reactivity and properties of the derived polymers have been revealed. Among the seven stereoisomers of HPMDA dianhydride revealed up to now [32], three species, including 1S,2R,4S,5R-HPMDA (H-PMDA or ccHPMDA), 1S,2S,4R,5R-HPMDA (H′-PMDA or ttHPMDA), and 1R,2S,4S,5R-HPMDA (H″-PMDA or ctHPMDA), have now been investigated as monomers in the research and development of high-performance PIs. It has been concluded that the stereoisomerism in the dianhydrides indeed affects the thermal and optical properties of the afforded PIs. Meanwhile, the polymerization reactivities of the different HPMDA dianhydrides are not only dependent on the steric structures of the dianhydrides, but also on the characteristics of the diamine monomers used. In the current work, the potential polymerization reactivities of the dianhydride and diamine monomers were roughly compared via theoretical simulation. The reactivity of the monomers was calculated according to the density functional theory (DFT)/B3LYP methods with Gaussian 09 software using the 6–311 G (d, p) basis set [33]. The lowest unoccupied molecular orbital (LUMO) energy levels (ε_LUMO_) for the HPMDA dianhydrides and the highest occupied molecular orbital (HOMO) energy levels (ε_HOMO_) for the fluorene-containing or 6FODA diamines were calculated, and the results are shown in Figure 1 and Figure 2, respectively. It was reported in the literature that the ε_LUMO_ value of one dianhydride and the ε_HOMO_ value of one diamine were closely related with their polymerization reactivity. A lower ε_LUMO_ value for the dianhydride and a higher ε_HOMO_ value for the diamine could roughly indicate the higher reactivity of the monomers [34]. According to Figure 1, H′-PMDA (ttHPMDA) with the lowest ε_LUMO_ value (−1.81 eV) might possess the highest reactivity among the three stereoisomers, which is in good agreement with the literature [31]. The reactivities of the three compounds decreased in the order of H′-PMDA (ttHPMDA) > H″-PMDA (ctHPMDA) > H-PMDA (ccHPMDA). With the same simulation procedure, the aromatic pyromellic dianhydride (PMDA) showed the ε_LUMO_ value of −4.17 eV, indicating a much higher polymerization reactivity than those of the alicyclic HPMDA. Thus, a high-temperature polycondensation procedure might be needed for the HPMDA-based PIs. As for the diamines, the reactivities of the three monomers decreased in the order of FDA (−5.39 eV) > FFDA (−5.59 eV) > 6FODA (−5.75 eV), according to the ε_HOMO_ values. These values are a bit lower than that of the common aromatic diamine of 4,4′-oxydianiline (ODA) (ε_HOMO_ = −5.08 eV), indicating the inferior reactivities of the fluorene-containing and fluoro-containing diamines. In the current work, ccHPMDA and ctHPMDA were chosen to polymerize with the three aromatic diamines, respectively.

**Figure 3:**
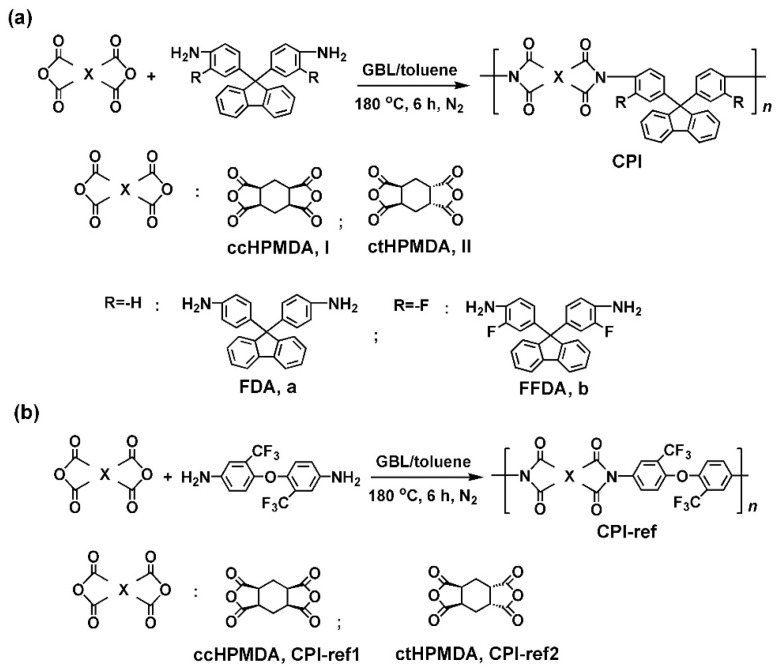
Preparation of CPI and the CPI-ref resins. (**a**) Fluorene-containing CPIs; (**b**) Referenced CPIs.

Polymerization was carried out according to the procedure shown in Figure 3. Due to the relatively lower reactivities of the utilized monomers, a high-temperature polymerization procedure was used to prepare the CPI resins. All the polymerization systems remained homogeneous, and no gelling or precipitation were observed, indicating the good solubility of the CPI resins in the reaction medium. This is mainly attributed to the non-conjugated alicyclic structures in the dianhydride units and the bulky fluorene or trifluoromethyl substituents in the diamine units. The inherent viscosities ([η]_inh_), molar mass and solubility of the CPI resins were measured, and the results are shown in Table 2. The entire resins exhibited moderate to high molar mass, with [η]_inh_ values higher than 0.51 dL/g and M_n_ values over 5.0 × 10^4^ g/mol. In addition, all the polymers showed PDI values lower than 1.70, indicating the minimal side reactions during the polymerization. For the fluorene-containing CPI resins, the ones derived from ctHPMDA showed higher M_n_ values than those derived from ccHPMDA. For example, CPI-II_a_ (ctHPMDA-FDA) had the M_n_ value of 11.41 × 10^4^ g/mol, which was much higher than that of CPI-I_a_ (ccHPMDA-FDA, M_n_ = 8.11 × 10^4^ g/mol). Meanwhile, the CPI resins based on FDA exhibited higher M_n_ values than those derived from FFDA. This is consistent with the polymerization reactivities’ prediction as shown in Figure 1 and Figure 2. However, with the 6FODA-based systems, the less reactive ccHPMDA afforded a resin with a higher M_n_ value, and the less reactive 6FODA also provided resins with high M_n_ values in the systems. As mentioned before, the molar masses of the final PIs are dependent on both the dianhydride and diamine monomers.

**Figure 4:**
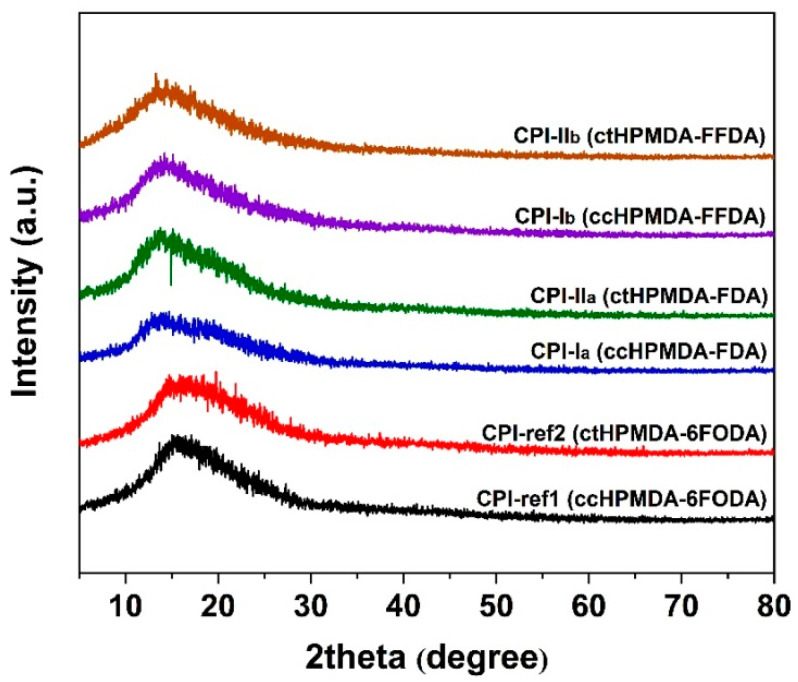
XRD spectra of CPI polymers.

All the CPI resins could be totally dissolved in the polar aprotic solvents, such as NMP and DMAc, at room temperature with a solid content of 10 wt. %. They were also soluble in less polar GBL. The fluorene-containing CPI resins were partially soluble in cyclopentanone (CPA) while the fluorine-containing CPI-ref1 and CPI-ref2 resins were totally soluble in CPA, indicating more obvious improvements of the solubility by the trifluoromethyl (–CF_3_) and flexible ether linkages (–O–) in the latter polymer systems. Basically, the good solubility of the current CPI resins was mainly attributed to the loose molecular chain packing and the deceased intra- and intermolecular interactions in the polymers, caused by the non-conjugated cyclohexane units in the dianhydride moiety and the bulky fluorene or –CF_3_ groups in the diamine moiety. Such structural features are clearly reflected by the XRD patterns of the polymers shown in Figure 4. All the polymers showed typical amorphous molecular structures, in which no clear crystalline regions were detected. This is quite beneficial to the penetration and diffusion of the organic solvents.

**Figure 5:**
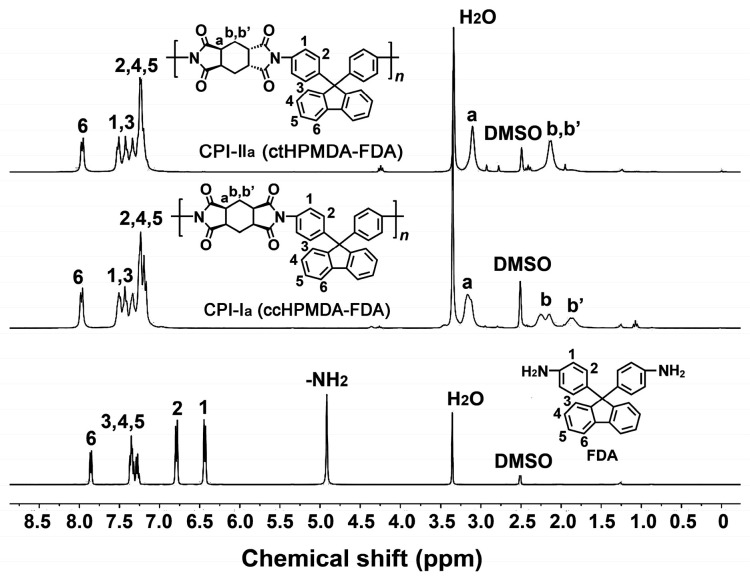
^1^H-NMR spectra of CPI resins based on FDA.

**Figure 6:**
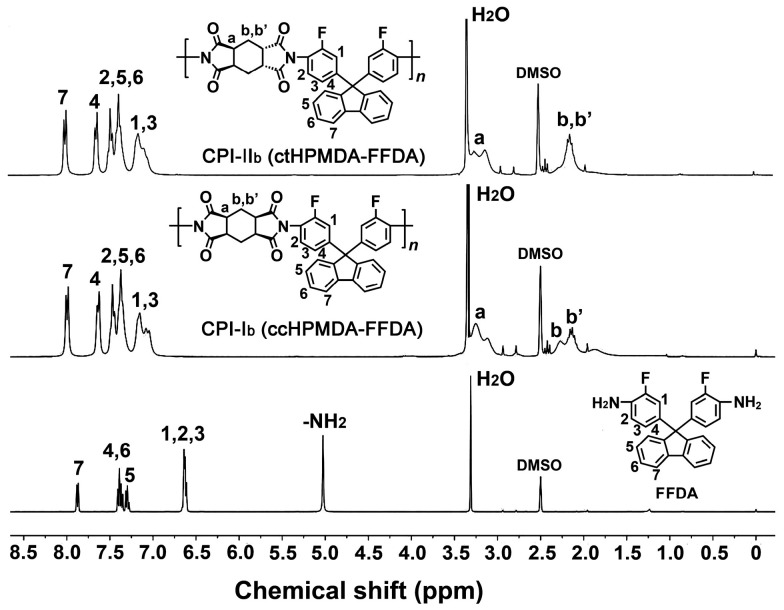
^1^H-NMR spectra of CPI resins based on FFDA.

The chemical structures of the CPI resins were confirmed by the ^1^H-NMR measurements and the spectra of the resins based on FDA and FFDA, and these, together with the assignments of every hydrogen proton, are shown in Figure 5 and Figure 6, respectively. Taking the CPI resins based on FDA as an example (Figure 5), it can be clearly observed that the characteristic absorption of the proton in –NH_2_ at 4.92 ppm completely disappeared in the spectra of the CPI resins. For the spectra of the polymers, the hydrogen proton absorptions were clearly divided into two parts, including the ones caused by the aromatic rings at the downfield areas and the ones due to the alicyclic rings at the upfield areas. The absorptions of H_6_ in the fluorene units appeared furthest downfield in the spectra. For the spectra of the ccHPMDA-based CPI-I_a_ and the ctHPMDA-based CPI-II_a_, the most obvious difference was the absorptions of H_b_ and H_b’_. For the ccHPMDA system, the H_b_ and H_b’_ protons exhibited split absorptions in the chemical shift range of 1.85~2.28 ppm, which might be due to the simultaneous actions of the two electron-withdrawing imide carbonyl groups. For the ctHPMDA system, the absorptions of H_b_ and H_b’_ protons overlapped, indicating the relatively weak effects of the two carbonyl groups. This is in good agreement with the structural features of the ctHPMDA dianhydride, in which the two imide rings showed opposite steric structures. For the CPI resins based on FFDA, the ^1^H-NMR spectra (Figure 6) revealed similar structural information. The information confirmed the successful preparation of the targeted CPI resins.

**Figure 7:**
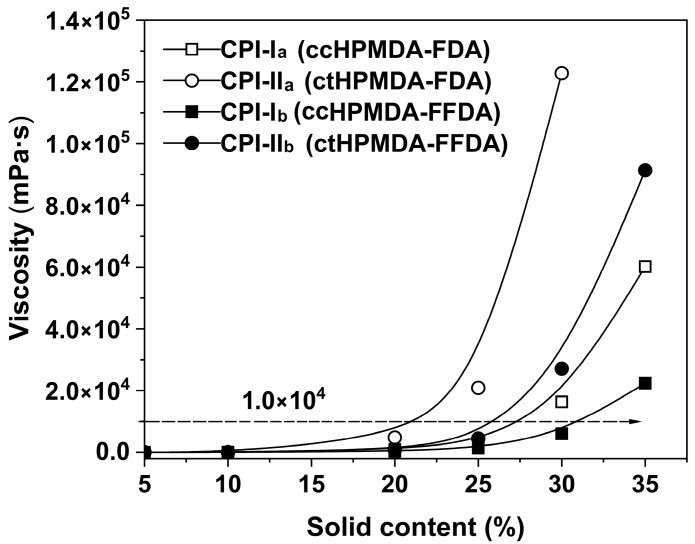
Viscosity–solid content relationship of CPI resin solution.

Although the current CPI resins were all soluble in the aprotic solvents, they showed different solubilities in the solvents, as evidenced by the viscosity–solid content plots of the resins in DMAc, shown in Figure 7. It is necessary to understand the change in viscosity of the CPI solutions with changes in solid contents, which helped us to choose the most suitable conditions for film preparation. Generally, in our work, an absolute viscosity around 1.0 × 10^4^ mPa·s for the CPI solution was used for the spin-coating fabrication of the CPI films in order to achieve a suitable film thickness at a specific spinning rate. As shown in Figure 7, to achieve this viscosity, the CPI solutions possessed increasing solid contents in the order of CPI-II_a_ < CPI-II_b_ < CPI-I_a_ < CPI-I_b_. This sequence is basically the opposite of the order of the M_n_ values of the resins. According to the results, different solid contents were chosen for CPI solution preparation. The excellent solubility of the current CPI resins endows them with good solution processability. Thus, a series of CPI films with desired thicknesses was successfully fabricated via the heat-curing of the preimidized CPI resin solution at elevated temperatures.

**Figure 8:**
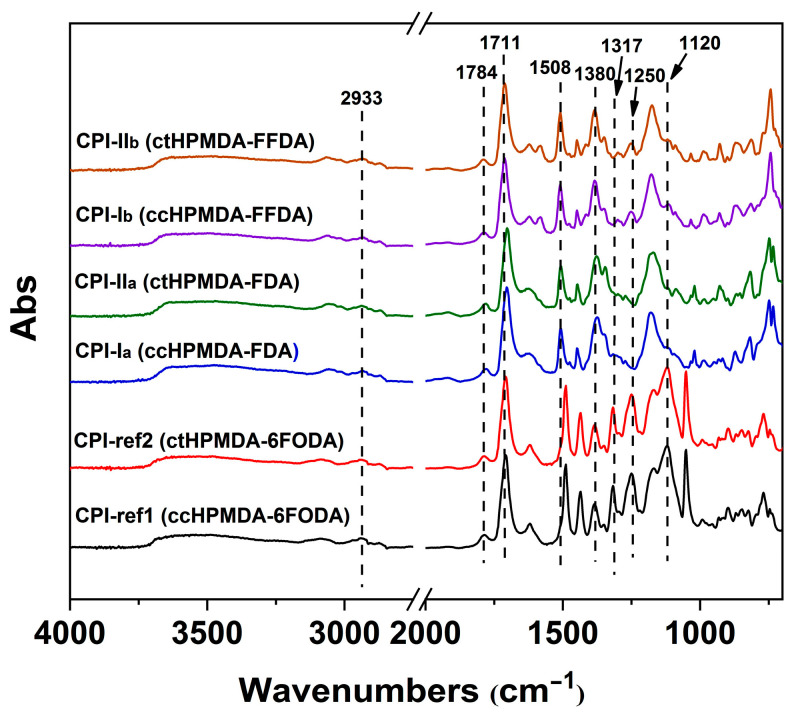
FTIR spectra of CPI films.

The chemical structures of the CPI films were further identified by FTIR measurements, as shown in Figure 8. Unlike the significantly different absorption peaks seen in the ^1^H-NMR spectra (Figure 5 and Figure 6) between the ccHPMDA-based and CtHPMDA-based PIs, a difference in absorption peaks due to the stereoisomerism of the structures was not observed in the FTIR spectra. All the polymers showed similar absorptions due to the similar chemical compositions. The similar parts include the characteristic absorptions of imide rings at ~1780 cm^−1^, ascribed to the asymmetrical carbonyl stretching vibrations, at ~1711 cm^−1^ due to the symmetrical carbonyl stretching vibrations, at ~1380 cm^−1^ caused by the C–N stretching vibrations, at 1508 cm^−1^ due to the stretching vibration of C=C in benzene rings in the diamine units, and at ~2933 cm^−1^ from the saturated C–H (–CH_2_–) absorptions in the dianhydride units. The differences mainly relate to the characteristic absorptions at ~1317 cm^−1^ due to the asymmetrical stretching of C–F bonds in –CF_3_ groups, and at 1120 cm^−1^ due to the –O– linkages, which were only detected in the spectra of the CPI-ref1 and CPI-ref2 polymers. In addition, symmetrical C–F stretching vibrations at ~1250 cm^−1^ were detected in the spectra of both the 6FODA-based CPI films and the FFDA-derived CPI films. The revealed structural features are highly consistent with those of the targeted CPI films.

### 3.2. Thermal Properties

**Figure 9:**
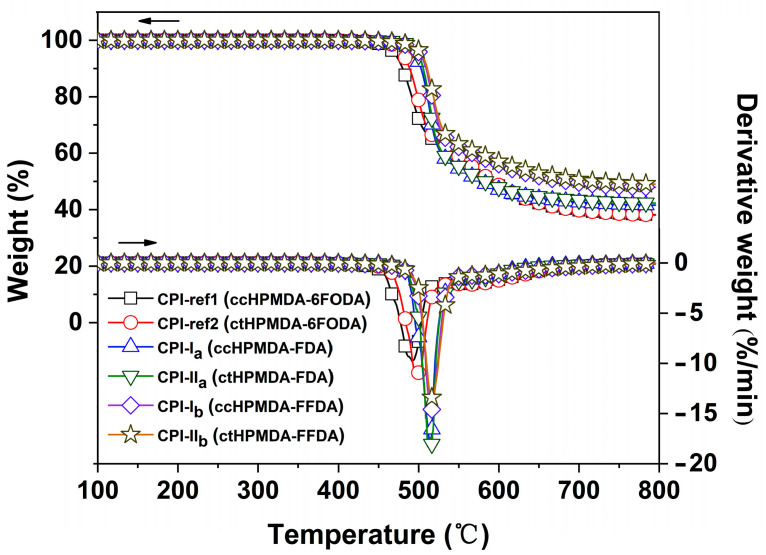
TGA and DTG curves of CPI films in nitrogen.

The fluorene-based polymers are well known for their high thermal stability due to the fused aromatic rings. Thus, it can be anticipated that the incorporation of the fluorene components into the thermally sensitive semi-alicyclic PIs might efficiently increase the thermal resistance of the derived polymers. First, the thermal decomposition behaviors of the CPI films were investigated by TGA and DTG tests, and the results are presented in Figure 9 and Table 3, respectively. It could be deduced from the Figure that all the CPI films maintained their initial weights before 450 °C, after which they began to decompose and showed 5% weight loss temperatures (T_5%_) in the range of 470.4~504.3 °C. The most rapid thermal decomposition occurred in the temperature range of 492.1~517.2 °C, and the films left 38.6~49.5 wt. % of their original weights at 750 °C in nitrogen. Basically, the FFDA-based CPI films exhibited the best thermal stability and the 6FODA-based CPI films showed the worst. For example, CPI-II_b_ (ctHPMDA-FFDA) showed T_5%_ and residual weight ratio at 750 °C (R_w750_) values of 504.3 °C and 49.5 wt. %, which are obviously higher than those related to CPI-ref2 (ctHPMDA-6FODA, T_5%_ = 480.6 °C; R_w750_ = 38.6 wt. %). This might be due to the higher aromatic ring contents in the fluorene-derived CPI films.

**Figure 10:**
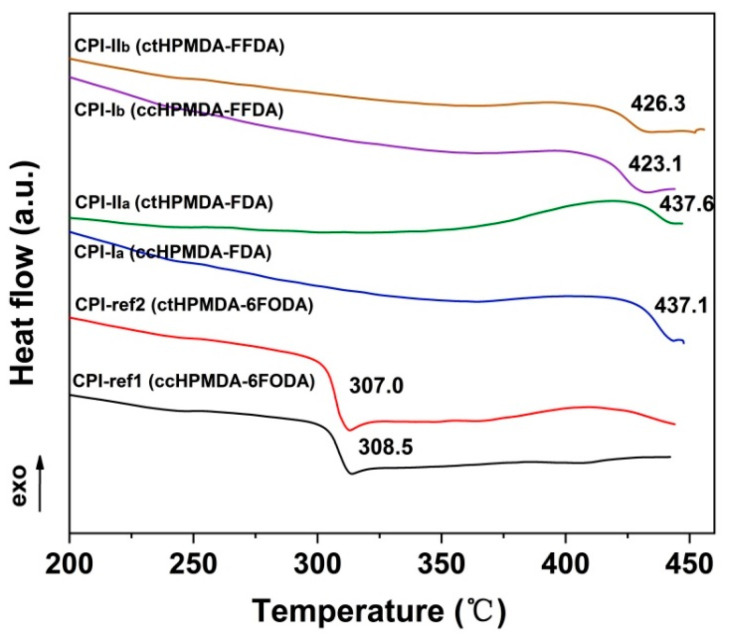
DSC curves of CPI films.

**Figure 11:**
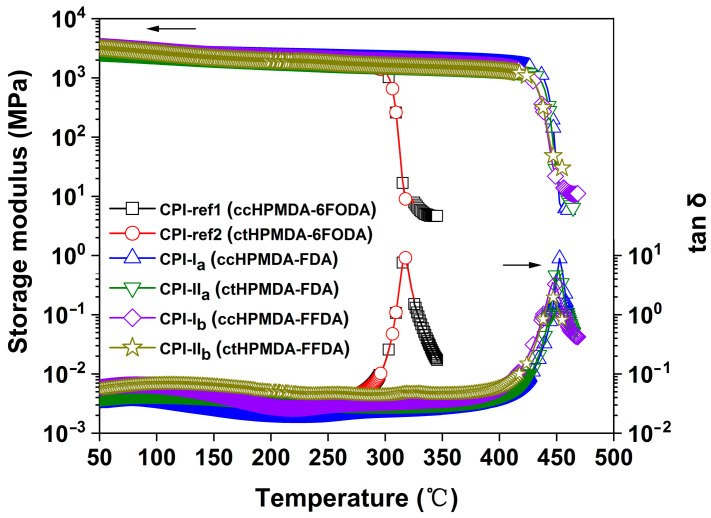
DMA curves of CPI films.

The glass transition temperatures (T_g_) of the CPI films were determined by DSC and DMA tests, and the results are shown in Figure 10 and Figure 11 and Table 4, respectively. According to the DSC plots shown in Figure 10, the CPI films exhibited clear glass transition behaviors in the tests. The fluorene-based CPI films showed T_g_ values that were more than 100 °C higher than those of the 6FODA-based ones. For example, the CPI-I_a_ (ccHPMDA-FDA) film had a T_g_ value of 437.1 °C, which is 128.6 °C higher than that of the analogous CPI-ref1 (ccHPMDA-6FODA) (T_g_ = 308.5 °C). The high-T_g_ feature of the fluorene-containing CPI films is mainly due to the non-planar structures in the FDA and FFDA diamine moieties caused by the bulky and rigid fluorene units, which efficiently increased the barrier against the free rotations and movements of the molecular chains at elevated temperatures. With respect to the effects of the stereoisomerism of the dianhydrides on the T_g_ values of the polymers, no obvious differences were observed for the ccHPMDA- and ctHPMDA-derived CPIs in the DSC measurements. Similar results were also observed in the DMA measurements, as shown in Figure 11. The T_g_ values of the polymers were identified as the peak temperatures of the tan δ plots. The fluorene-based CPI films exhibited T_g_ values in the range of 446.0~453.2 °C, which were also obviously higher than those of the 6FODA-based polymers (T_g_ ≈ 317 °C). In addition, the fluorene-containing CPI films could maintain most of their initial storage modulus up to 400 °C, while the modulus of the 6FODA-derived ones dramatically dropped when the temperature was higher than 300 °C. This also reflects the superior thermal stability of the former polymers.

**Figure 12:**
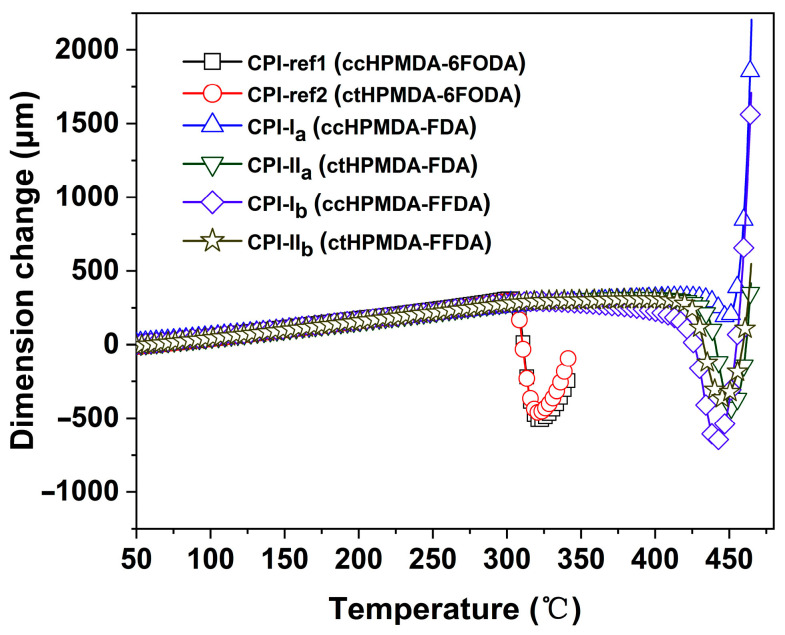
TMA curves of CPI films.

The high-temperature dimensional stability of the CPI films was evaluated by TMA measurements and the results are shown in Figure 12. The fluorene-containing CPI films showed slight thermal expansion before 400 °C and then shrunk sharply when the test temperature further increased, indicating the occurrence of the glass transition behaviors and the ordered rearrangements of the molecular chains. When this course finished, the CPI films expanded again, and their dimension change showed a nearly linear increasing trend. For the fluoro-containing CPI-ref1 and CPI-ref2 systems, this phenomenon occurred at around 300 °C, indicating the inferior thermal resistance of the polymers. In the temperature range of 50~250 °C, the fluorene-based CPI films showed linear coefficients of thermal expansion (CTE) values of 57.4 × 10^−6^/K~66.5 × 10^−6^/K, which were comparable to those of the 6FODA-based films (60.7 × 10^−6^/K for CPI-ref1 and 62.2 × 10^−6^/K for CPI-ref2). The CPI films derived from ctHPMDA showed somewhat higher CTE values than those based on the analogous ccHPMDA. This might be due to the relatively looser packing of molecular chains in the ctHPMDA-PI systems, which arose from the steric effects of the dianhydride. The higher CTE values for the semi-alicyclic CPI films have been cited as one of the main defects in relation to applications in advanced optoelectronic fields. This is mainly ascribed to the non-conjugated and less interactive molecular chain structures in the cyclohexane dianhydride units. Fortunately, many methodologies are available that could be applied to reducing the CTE values of the semi-alicyclic CPI films. Related studies have been widely reviewed in the literature [35,36].

### 3.3. Optical Properties

It is well established that high optical transparency, low yellow indices, low haze, low birefringence, and low optical retardation, together with high T_g_ and low CTE, are often prerequisites for optical polymer films used as components in advanced flexible display devices. It is indeed challenging to achieve all of the property requirements in one polymer. As discussed above, the fluorene-based CPI films could provide good thermal stability. Thus, the optical properties of the polymer films were further investigated.

**Figure 13:**
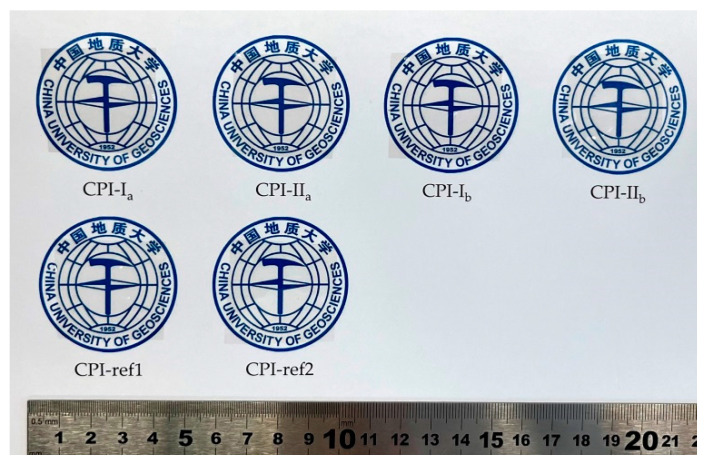
Appearances of the fluorene-containing and referenced CPI films.

**Figure 14:**
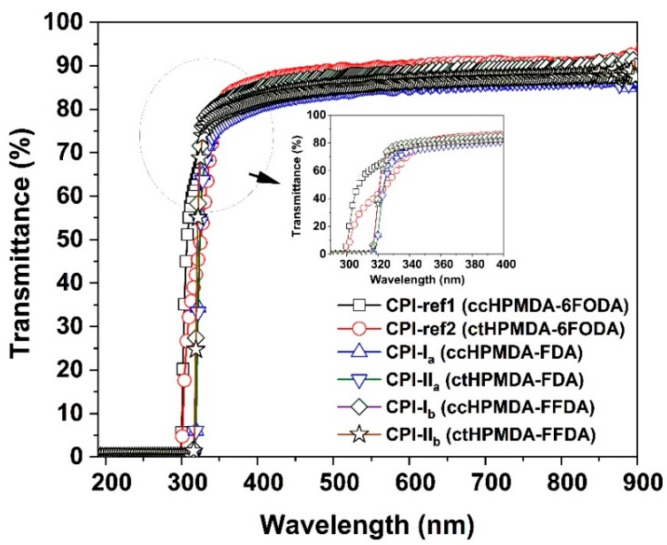
UV-Vis spectra of CPI films.

**Figure 15:**
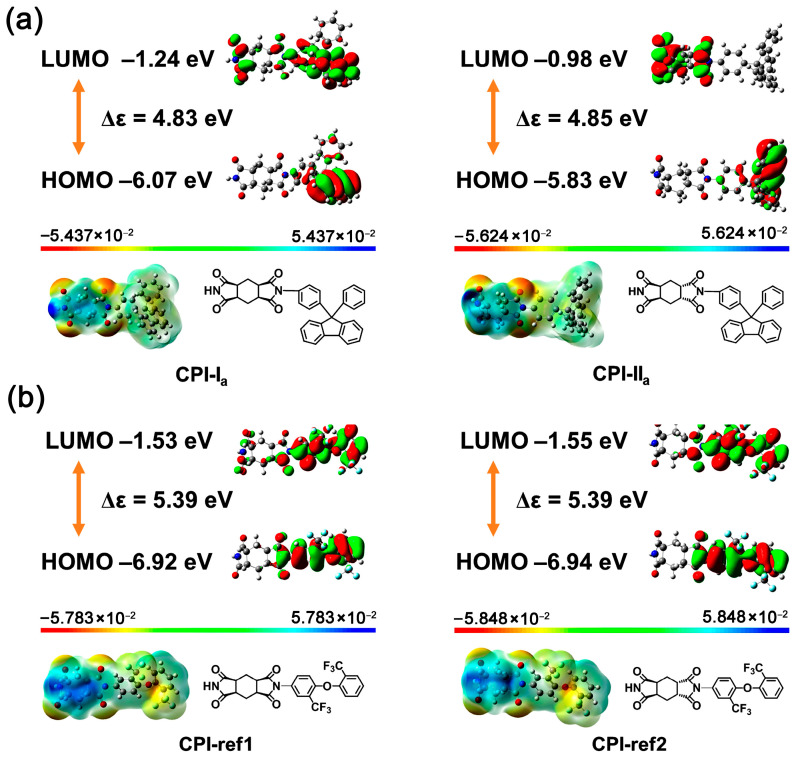
Molecular orbit energy levels of the CPI films. (**a**) FDA-based; (**b**) 6FODA-based.

**Figure 16:**
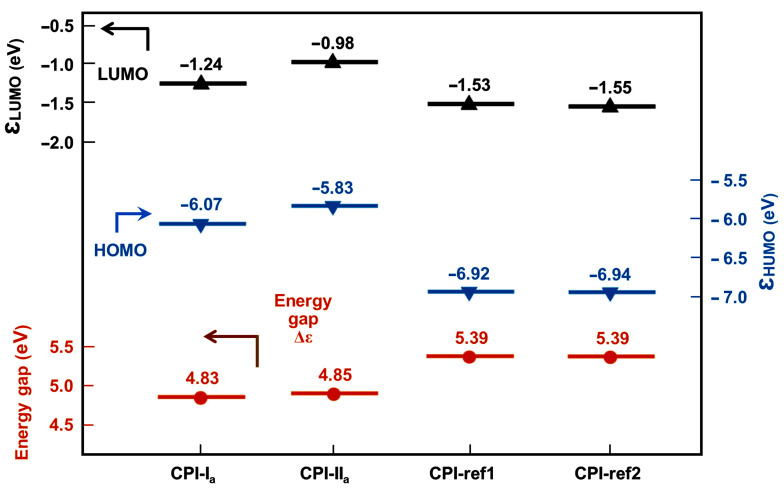
Comparison of energy level gap (Δε) values of the CPI films.

First, the appearances of the developed fluorene-containing and referenced CPI films are shown in Figure 13. All the CPI films exhibited very pale colors and good optical transparency. Figure 14 presents the UV-Vis spectra of the CPI films and the optical data are tabulated in Table 4. The CPI films showed UV cutoff wavelengths (λ_cut_) in the range of 287~304 nm, indicating good optical transparency in the visible light region (wavelength: 400~760 nm). The optical transmittances of the CPI films at 400 nm (T_400_) and 450 nm (T_450_) were all higher than 80% at the thickness of 20 μm. Basically, the fluorine-containing 6FODA-PI films exhibited higher optical transmittances than those of the fluorene-PI counterparts. For example, the CPI-ref1 (ccHPMDA-6FODA) film showed a T_400_ value of 84.9%, which is higher than those of analogous CPI-I_a_ (ccHPMDA-FDA, T_400_ = 82.1%) and CPI-I_b_ (ccHPMDA-FFDA, T_400_ = 84.6%). In theory, the existence of the highly electronegative –CF_3_ groups efficiently reduced the charge transfer (CT) actions from the electron-donating diamine units to the electron-withdrawing dianhydride units, resulting in the prohibition of visible light absorption by the polymers. Meanwhile, the non-conjugated cyclohexane rings also caused a reduction in the CT effects. The CT interactions could be explained by calculating the frontier molecular orbital (MO) energies of the CPIs, as shown in Figure 15. The calculated ε_HOMO_, ε_LUMO_, and energy gap (Δε, |ε_HOMO_ − ε_LUMO_|) values are shown in Figure 16. In the literature, both experimental and theoretical investigations of the PI films have proven that intra- and intermolecular CT with diamine to dianhydride moiety can play an important role in determining the ultraviolet absorption of PI films [37,38]. The Δε values of the CPI films could be used to roughly reflect the difficulties related to CT interactions within the molecular chains of the PIs. The larger this value is, the more difficult it will be for it to cause CT interactions. The Δε values of the CPI films increased in the order of CPI-I_a_ (4.83 eV) < CPI-II_a_ (4.85 eV) < CPI-ref1 (5.39 eV) = CPI − ref2 (5.39 eV). Thus, CPI-ref1 and CPI-ref2 showed the best optical transparency among the CPI systems.

Secondly, the CIE Lab optical parameters of the CPI films were measured and compared in Table 4. All the CPI films showed low yellow indices (b*) in the range of 0.60~1.01 and low haze values below 3.0%. As anticipated, the CPI films derived from 6FODA exhibited the lowest yellow indices and the highest lightness (L*) in the series of films. In addition, the stereoisomerism of the dianhydrides showed minimal effects on the color parameters of the CPI films.

Lastly, the refractive indices (n_TE_ and n_TM_), birefringence, and optical retardations of the CPI films were tested, and the results are tabulated in Table 4. It is well known that the refractive index of a polymer film is dependent on the sum of the molar refraction (P) to the molar volume (V) of each group in its molecular structure, according to the Lorentz–Lorenz equation [39]. The lower the P/V value, the lower the refractive index of the polymer film, and vice versa. The specific values of the P and V parameters of common substituents have been established in the literature via experimental or calculation methods [40]. Fluorine-containing groups are usually listed among the low-P substituents, while fluorene-containing groups are listed among the high-P and high-V components [41]. These structural features explain the lower refractive indices of the current 6FODA-based CPI films compared with those of the fluorene-based ones. For the CPI films based on ccHPMDA, the n_av_ values increased in the order of CPI-ref1 (ccHPMDA-6FODA, 1.5407) < CPI-I_b_ (ccHPMDA-FFDA, 1.6094) < CPI-I_a_ (ccHPMDA-FDA, 1.6296). The ctHPMDA-derived CPI film showed a similar trend. The fluorinated 6FODA-PI and FFDA-PI films showed lower n_av_ values than those of the non-fluorinated FDA-PI films due to the low molar refraction of the fluorine-containing groups (–F or –CF_3_). The fluorene-containing CPI films show very low birefringence (Δn) values at the level of minus fourth power of ten, and further, exhibit quite low optical retardations below 10 nm. The R_th_ values for the current fluorene-based CPI films (thickness: 10 μm) were obviously lower than those of the CPI systems reported in the literature, such as the wholly aromatic ones in [42] (R_th_: 114~172 nm, measured with a film thickness of 2 μm), the ones in [43] (R_th_: 84.5~135 nm, measured with a film thickness of 20 ± 5 μm), and the ones in [21] (R_th_: 42~279 nm, measured with a film thickness of 15~18 μm), and were also lower than those of the semi-alicyclic ones in [44] (R_th_: 276~496 nm, measured with a film thickness of 10 μm). The excellent optical properties of the current CPI films benefited partly from the fluorene substituents in the diamine units, and also from the cyclohexane structure in the dianhydride moiety. On one hand, the bulky fluorene groups efficiently prohibited the folding of the molecular chains in one direction in the CPIs, providing the low optical anisotropies of the polymers [45]. On the other hand, the non-conjugated cyclohexane rings in the dianhydride units increased the molecular chain mobility and decreased the chain packing density, resulting in the low Δn values of the CPI films. This also explains the low R_th_ feature of CPI-ref1 and CPI-ref2, although they showed slightly higher R_th_ values than those of their fluorene-based counterparts.

## 4. Conclusions

Functional CPI films with high T_g_, high optical transparency, low b*, low haze, low Δn and low R_th_ values were designed and successfully developed based on semi-alicyclic and fluorene chemistry. The CPI-I_a_ film derived from ccHPMDA and FDA showed the best combined properties, including a T_400_ value of 82.1%, a b* value of 1.01, a haze value of 1.3%, a T_g_ value of 437.1 °C, and a R_th_ value of 2 nm. In addition, the CPI-I_a_ film showed a CTE value of 57.4 × 10^−6^/K in the temperature range of 50~250 °C, which is a bit higher than that required in some specific optoelectronic applications. The incorporation of inorganic nanoparticles might be an efficient way to reduce the CTE values of the fluorene-based CPI films. Detailed research works are being performed in our laboratory, and will be reported in the near future.

## Figures and Tables

**Table 1:** Formula for the CPI synthesis.

PI	ccHPMDA (g, mol)	ctHPMDA (g, mol)	FDA (g, mol)	FFDA (g, mol)	GBL (g)
CPI-I_a_	22.417, 0.1	NA	34.844, 0.1	NA	171.8
CPI-I_b_	22.417, 0.1	NA	NA	38.442, 0.1	171.8
CPI-II_a_	NA ^a^	22.417, 0.1	34.844, 0.1	NA	171.8
CPI-II_b_	NA	22.417, 0.1	NA	38.442, 0.1	171.8

^a^ Not applicable.

**Table 2:** Inherent viscosities, molar mass and solubility of CPI resins.

PI	[η]_inh_ ^a^ (dL/g)	Molar Mass ^b^			Solubility ^c^				
		M_n_ (×10^4^ g/mol)	M_w_ (×10^4^ g/mol)	PDI	NMP	DMAc	GBL	CPA	THF
CPI-I_a_	0.68	8.58	12.92	1.51	++	++	++	+	+
CPI-II_a_	0.83	11.41	17.95	1.57	++	++	++	+	+
CPI-I_b_	0.51	5.04	8.20	1.63	++	++	++	+	+
CPI-II_b_	0.62	7.09	11.71	1.65	++	++	++	+	+
CPI-ref1	1.03	13.77	20.90	1.52	++	++	++	++	+
CPI-ref2	0.96	9.74	15.76	1.62	++	++	++	++	+

^a^ Inherent viscosities measured with a 0.5 g/dL PI solution in NMP at 25 °C. ^b^ M_n_: number average molar mass. M_w_: weight average molar mass. PDI: dispersity index, PDI = M_w_/M_n_. ^c^ ++: Soluble. +: partially soluble. insoluble. GBL: γ-butyrolactone. CPA: cyclopentanone. THF: tetrahydrofuran.

**Table 3:** Thermal properties of CPI films.

Samples	T_g, DSC_^a^ (°C)	T_g, DMA_^a^ (°C)	T_5%_^a^ (°C)	T_max_^a^ (°C)	R_w750_^a^ (%)	CTE ^a^ (×10^−6^/K)
CPI-I_a_	437.1	453.2	496.2	514.6	41.8	57.4
CPI-II_a_	437.6	449.5	499.0	514.9	42.7	66.5
CPI-I_b_	423.1	446.0	501.9	516.9	48.4	60.4
CPI-II_b_	426.3	447.0	504.3	517.2	49.5	63.5
CPI-ref1	308.5	317.9	470.4	492.1	38.7	60.7
CPI-ref2	307.0	317.8	480.6	497.4	38.6	62.2

^a^ T_g, DSC_: Glass transition temperatures according to the DSC measurements. T_g, DMA_: Glass transition temperatures according to the DMA measurements (peaks of tanδ plots). T_5%_: Temperatures at 5% weight loss. T_max_: Temperatures at the most rapid thermal decomposition rate. R_w750_: Residual weight ratio at 750 °C in nitrogen. CTE: linear coefficient of thermal expansion in the range of 50–250 °C.

**Table 4:** Optical properties of CPI films.

Samples	λ_cut_^a^ (nm)	T_400_^a^ (%)	T_450_^a^ (%)	n_TE_^a^	n_TM_^a^	n_av_^a^	Δn ^a^	R_th_^a^ (nm)	L* ^a^	a* ^a^	b* ^a^	Haze (%)
CPI-I_a_	303	82.1	84.5	1.6296	1.6298	1.6296	0.0002	2	95.24	0.01	1.01	1.3
CPI-II_a_	304	81.0	83.2	1.6306	1.6315	1.6309	0.0009	9	95.27	−0.02	0.86	2.8
CPI-I_b_	298	84.6	86.4	1.6091	1.6100	1.6094	0.0009	9	95.56	−0.01	0.76	1.2
CPI-II_b_	299	82.2	84.0	1.6119	1.6127	1.6122	0.0008	8	95.52	0.01	0.67	2.1
CPI-ref1	287	84.9	86.7	1.5419	1.5382	1.5407	0.0037	37	96.28	−0.01	0.60	1.8
CPI-ref2	293	86.3	87.9	1.5432	1.5371	1.5412	0.0061	61	96.26	−0.01	0.65	0.6

^a^ λ_cut_: Cutoff wavelength. T_400_, T_450_: Transmittance at the wavelengths of 400 nm and 450 nm with a thickness of 20 μm, respectively. n_TE_, n_TM_: In-plane and out-of-plane refractive indices of the CPI films, respectively. n_av_: Average refractive indices of the CPI films. Δn: Birefringence, Δn = n_TE_ − n_TM_. R_th_: Optical retardation, R_th_ = Δn × d, d = 10 μm. L*, a*, b*, see Measurements part.

## Data Availability

Data are contained within the article.
